# 0.16 µm–BCD Silicon Photomultipliers with Sharp Timing Response and Reduced Correlated Noise

**DOI:** 10.3390/s18113763

**Published:** 2018-11-03

**Authors:** Mirko Sanzaro, Fabio Signorelli, Paolo Gattari, Alberto Tosi, Franco Zappa

**Affiliations:** 1 Dipartimento di Elettronica, Informazione e Bioingegneria, Politecnico di Milano, I-20133 Milan, Italy; mirko.sanzaro@polimi.it (M.S.); fabio.signorelli@mail.polimi.it (F.S.); alberto.tosi@polimi.it (A.T.); 2 Technology R&D, STMicroelectronics, I-20041 Agrate Brianza, Italy; paolo.gattari@st.com

**Keywords:** Silicon photomultiplier (SiPM), photon counting, time-correlated single-photon counting (TCSPC), photon number resolution, afterpulsing, optical crosstalk

## Abstract

Silicon photomultipliers (SiPMs) have improved significantly over the last years and now are widely employed in many different applications. However, the custom fabrication technologies exploited for commercial SiPMs do not allow the integration of any additional electronics, e.g., on-chip readout and analog (or digital) processing circuitry. In this paper, we present the design and characterization of two microelectronics-compatible SiPMs fabricated in a 0.16 µm–BCD (Bipolar-CMOS-DMOS) technology, with 0.67 mm × 0.67 mm total area, 10 × 10 square pixels and 53% fill-factor (FF). The photon detection efficiency (PDE) surpasses 33% (FF included), with a dark-count rate (DCR) of 330 kcps. Although DCR density is worse than that of state-of-the-art SiPMs, the proposed fabrication technology enables the development of cost-effective systems-on-chip (SoC) based on SiPM detectors. Furthermore, correlated noise components, i.e., afterpulsing and optical crosstalk, and photon timing response are comparable to those of best-in-class commercial SiPMs.

## 1. Introduction

Silicon photomultipliers (SiPMs) are dense arrays of Single-Photon Avalanche Diodes (SPADs) [1], each one with its own quenching resistor, connected in parallel [2]. The output is a current pulse, whose amplitude is proportional to the number of SPADs that are simultaneously ignited. 

SiPMs are widely used in applications where scintillation light has to be detected with good timing resolution, e.g., in high-energy physics experiments [3] and positron emission tomography (PET) [4]. Additionally, SiPMs are suitable sensors when single-photon sensitivity and large collection area are required, such as in near-infrared spectroscopy (NIRS) [5] and three-dimensional imaging (LIDAR–light detection and ranging) [6].

Commercially-available SiPMs exploit custom fabrication technologies for optimizing their performance. However, efforts are underway to fabricate SiPMs in CMOS [7] and BCD [8] technologies, aiming at cost-effective systems-on-chip (SoC) based on SiPM detectors. Finally, digital SiPMs (dSiPMs), where each pixel integrates an active quenching circuit and provides a digital output to on-chip digital processing electronics, have been demonstrated [9]. Despite their lower fill-factor, dSiPMs offer several very attractive features. Among them is the possibility of switching off the noisiest SPADs.

In [10] we reported on SPADs we developed in a 0.16 µm–BCD technology, attaining more than 60% photon detection efficiency (PDE) at 500 nm, dark count rate (DCR) density lower than 0.2 cps/µm^2^, and less than 30 ps FWHM (full-width at half maximum) timing jitter. In this paper, we present two SiPMs based on BCD SPADs. 

The rest of the paper is organized as follows: Section 2 presents the BCD SiPM microcell; Section 3 reports an experimental characterization of BCD SiPMs; Section 4 concludes the paper with a performance comparison between BCD SiPMs and some commercial ones.

## 2. Materials and Methods

**Figure 1:**
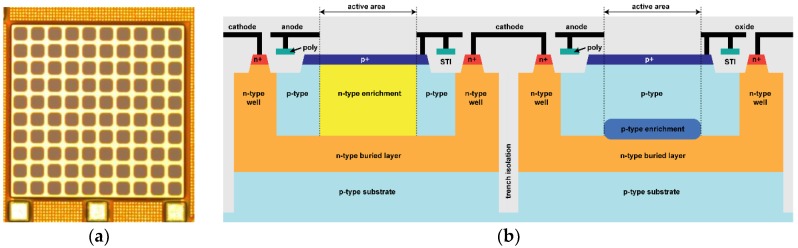
(**a**) Micrograph of a BCD SiPM with 10 × 10 microcells; (**b**) Cross-section of a BCD shallow SiPM microcell (left pixel) and of a deep SiPM microcell (right pixel).

We designed SiPMs with 10 × 10 square microcells, a total area of 0.45 mm^2^, and with 67 μm pitch between adjacent cells. Each SPAD has a square active area (50 µm side length) with rounded corners (12.5 µm radius). The resulting fill-factor (FF), i.e., the ratio between the active area and the total area of the sensor, is 53%. Figure 1a shows a micrograph of the BCD SiPM. The non-sensitive region between adjacent active areas is shielded from light by a metal layer.

The SiPM cross-section is shown in Figure 1b. Each SPAD is fully enclosed in a double-well pocket, formed by a n-type buried layer, for isolation from the p-type substrate, and a heavily doped n-type well, which provides a low resistance path to the cathode contact. This fabrication technology also features deep trenches, which are exploited for electrical and optical isolation between cells.

A custom implant, referred to as enrichment in the following, defines the SPAD active area. The enrichment doping can be either a low energy phosphorus implant, as in Figure 1b (left pixel), or a high energy boron implant, as in Figure 1b (right pixel), leading to different depths of the avalanche region [10]. In the former case, the avalanche region is close to the silicon surface, as in a typical p^+^/n CMOS SPAD, so we named the device a ‘shallow’ SPAD. In the latter case, the device was named ‘deep’ SPAD, because the avalanche region of the resulting p/n^+^ SPAD is close to the n-type buried layer. Hence, as shown in [10], different devices can be fabricated from the same masks set by means of split lots. In this work, we present two designs: (i) a shallow SiPM, with a breakdown voltage (V_BD_) of 25.4 V at room temperature, and (ii) a deep SiPM with V_BD_ = 26.3 V.

Even though we added custom implants to the BCD process flow-chart, the thermal budget was not altered. As a result, the developed SiPMs maintain full compatibility with Bipolar, CMOS and DMOS on-chip circuitry, thus being suitable for effective SoC integration.

A polysilicon resistor (with a resistance higher than 400 kΩ at room temperature) is integrated along the four sides of the SPAD. Indeed, the quenching resistor replaces the polysilicon field-plate that avoids edge breakdown in [10]. Finally, a metal ring connected to the output pad is laid out above the resistor for increasing the so-called “quenching capacitance” (see also [11]). This produces a higher peak and a faster rise time in the output current signal [12], eventually reducing the timing jitter.

The readout circuit employed in the experimental characterization is based on a trans-impedance amplifier (TIA) connected to the anode terminal. Specifically, we employed an Analog Devices Inc. AD8000 current feedback amplifier (CFA) and a 1 kΩ feedback resistor. Thanks to the low inverting input impedance of the CFA, the TIA is stable with no need for a feedback capacitor. The CFA output drives a double-end terminated 50 Ω coaxial cable. The printed circuit board (PCB) size is 25 mm × 20 mm, small enough to be housed inside a cryostat head for low temperature characterization.

## 3. Results

We thoroughly characterized the most important features of the fabricated BCD SiPMs: pulse amplitude spectrum, PDE, DCR, crosstalk probability, afterpulsing and timing response. Unless otherwise stated, all measurements have been performed at a temperature of 300 K.

### 3.1. Pulse Amplitude Spectrum

Pulse amplitude distribution is important in applications exploiting the photon number resolving capability of SiPMs. We characterized the pulse amplitude spectrum by illuminating the SiPMs with a pulsed laser at 850 nm, 1 MHz repetition rate. The amplified SiPM output and the laser synchronization signal are fed to a Tektronix 4104B oscilloscope (1 GHz–bandwidth, 5 GSample/s). With the oscilloscope synchronized to the laser, we recorded hundreds of waveforms and built the histogram of pulse heights.

**Figure 2:**
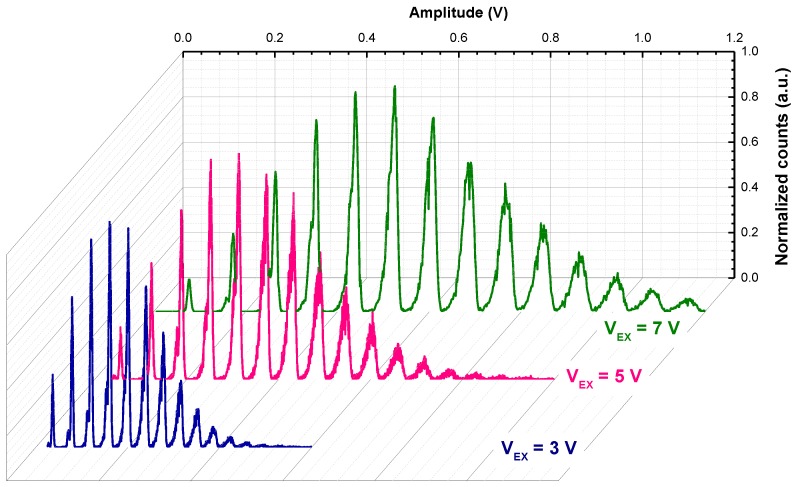
Pulse amplitude histogram for a deep SiPM operated at different excess bias voltages under the same illumination condition.

Figure 2 shows the pulse amplitude spectrum for a deep SiPM operated at different excess bias voltages, V_EX_ (i.e., the voltage above breakdown). Histograms show a very good peak separation thanks to the good uniformity of the chosen BCD technology and the low correlated noise (i.e., afterpulsing and delayed crosstalk), which increases the peak-to-valley ratio [13]. The average number of detected photons per pulse increases with excess bias, due to related increase of PDE and crosstalk.

**Figure 3:**
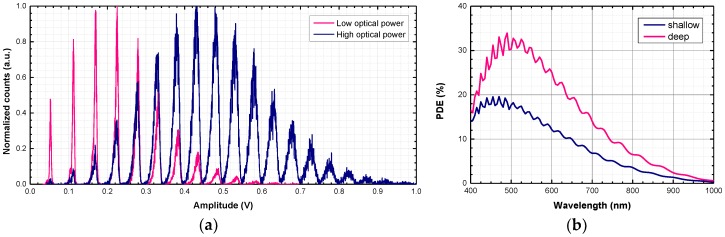
(**a**) Pulse amplitude histogram for a shallow SiPM at 5 V excess bias under pulsed illumination; (**b**) PDE vs. wavelength of the two developed SiPMs at 5 V excess bias (FF included).

The same plot for a shallow SiPM at two different optical powers is shown in Figure 3a. By increasing the illumination power (i.e., the photon flux), the distribution shifts toward higher amplitudes and more peaks become visible at the right-hand side of the spectrum. Up to 18 simultaneous photon absorptions can be clearly distinguished in Figure 3a.

### 3.2. Photon Detection Efficiency

The overall PDE has been computed by multiplying the PDE of BCD SPADs [10], as measured from discrete devices on the same wafers, by the SiPM FF (53%):

PDE_SiPM_ = PDE_SPAD_ · FF. (1)

In this way, we avoid affecting the measurement by correlated noise [13]. The calculated spectrally resolved PDE of both shallow and deep SiPMs is shown in Figure 3b. Owing to the higher avalanche triggering probability of electrons compared to holes [14], the deep SiPM leads to higher sensitivity compared to shallow SiPMs [10]. The PDE peak of the deep SiPM is 29%, 34% and 36% at 490 nm, when the device is biased at 3 V, 5 V and 7 V excess bias, respectively. At V_EX_ = 5 V, excess bias, the PDE is still 4% at 850 nm.

### 3.3. Noise Characterization

**Figure 4:**
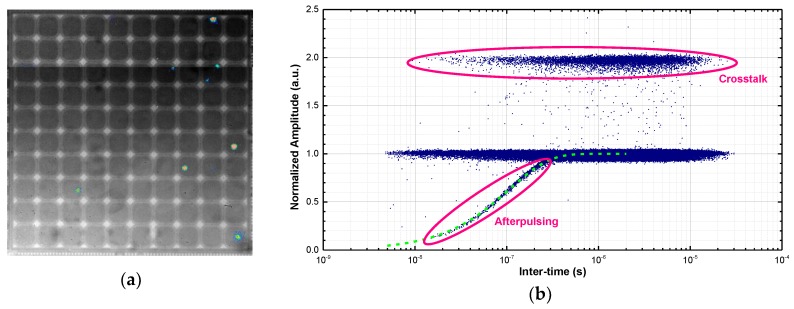
(**a**) Near-infrared emission from the back side of a shallow SiPM chip at V_EX_ = 8 V, as acquired by an InGaAs camera; (**b**) Scatter plot of timing and amplitude of secondary pulses for a shallow SiPM at 5 V of excess bias. The pulse amplitude is normalized to the average height of a single cell signal. The exponential fitting of the recovery transient is also reported.

The DCR of a SiPM cannot be computed by simply multiplying the typical DCR of a SPAD developed in the same technology by the number of microcells of the SiPM. The reason being that hot pixels give the highest contribution, as demonstrated by the emission microscopy (EMMI) image shown in Figure 4a. Such optical analysis technique detects the faint near-infrared (NIR) emission from all SPADs in a dark environment, eventually localizing defective ones (~7% in this sample). It is worth noting that the avalanche is promptly quenched by the resistor in each cell and does not propagate over the entire SPAD. As a result, hot spots mark the actual location of microplasmas.

We characterized the device noise by following a procedure similar to the one described in [15]: we digitized hundreds of 2 ms-long waveforms and, after time-stamping all avalanche pulses, we built a scatter plot in which each point is the normalized amplitude of the secondary pulse as a function of the time interval between that pulse and the previous one. We employed the filtering technique described in [16] to shorten pulse duration to few nanoseconds, thus avoiding height digitization errors due to piled-up events. The inter-times triggered by pulses with normalized amplitude higher than 1.05 or lower than 0.95 have been discarded as in [16].

An example of this measurement is shown in Figure 4b, where different noise contributions are highlighted:

- ‘primary’ dark counts are events with unitary amplitude;
- events with double amplitude are crosstalk events triggered by a dark count;
- afterpulses occur at short inter-times (< 300 ns in our case) and their amplitude follows the exponential recovery of the microcell.

It is worth noting that ‘delayed’ crosstalk (DeCT) events (i.e., photons emitted by a pixel, absorbed in the substrate and triggering a neighboring pixel owing to the delayed diffusion of the photogenerated carrier) are not visible. The reason being that junction isolation between each pixel and the underlying substrate prevents carrier diffusion from the substrate to the SPAD depleted region [17].

**Figure 5:**
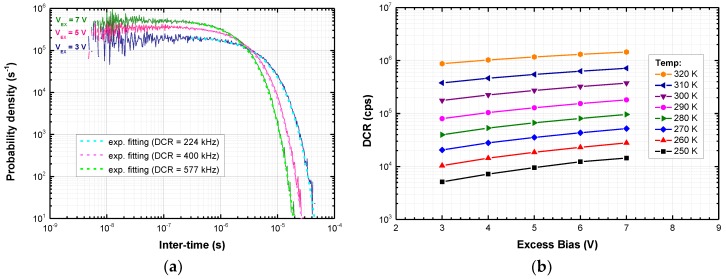
(**a**) Inter-time probability density for a shallow SiPM: the primary DCR is the inverse of the exponential fitting time constant; (**b**) Primary DCR of a deep SiPM, as a function of excess bias, at different temperatures.

The plot in Figure 5a is obtained from the scatter plot of Figure 4b. First, the time axis is divided in time bins and the number of secondary events (regardless their amplitude) falling into each bin is calculated. Then, each bin of the resulting histogram is normalized to the total number of events and is divided by its time-width, in order to obtain the probability density that describes the inter-times between consecutive pulses. Finally, the primary DCR is extrapolated by fitting the late part of the histogram (> 300 ns) with an exponential decay. 

This analysis has been repeated at different excess bias voltages and temperatures. The DCR as a function of excess bias at different temperatures is shown in Figure 5b. The breakdown drift over temperature (~30 mV/K) has been compensated. For both devices, the DCR doubles every 10 K. At 250 K, a temperature easily attainable by means of a TE-cooler, DCR is below 10 kcps at V_EX_ = 5 V.

Deep trenches surrounding each microcell reflect most secondary photons propagating from one detector to another through a direct optical path, thus strongly reducing optical crosstalk. However, since trenches are neither coated by metal nor filled with an absorptive/opaque material, the reflection is not 100% effective. Furthermore, secondary photons reflected at the SiPM chip backside can reach a neighboring microcell via indirect paths [18].

**Figure 6:**
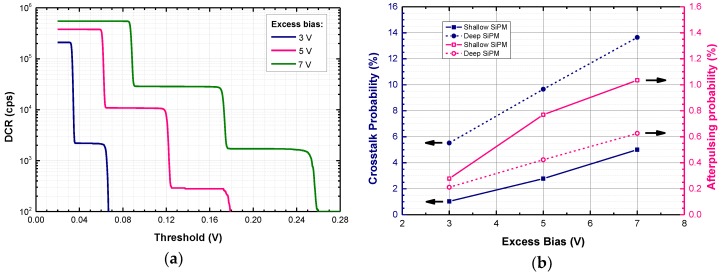
(**a**) Room temperature DCR as a function of discrimination threshold for a shallow SiPM at different excess bias; (**b**) Crosstalk and afterpulsing probability of shallow and deep SiPMs as a function of excess bias.

Figure 6a shows the DCR at different discriminator thresholds, calculated as the total number of pulses in the analyzed waveforms crossing a given threshold, divided by the integration time. From the plot, it is possible to compute the optical cross-talk probability as:

Crosstalk = DCR_2p_/DCR_1p_, (2)

where DCR_2p_ and DCR_1p_ are the DCR values measured with the discriminator threshold set at 1.5 and at 0.5 times the height of a single-cell avalanche, respectively. Figure 6b reports the crosstalk probability as a function of excess bias. At V_EX_ = 5 V, we obtained 2.8%, and 9.7% for the shallow and deep SiPM, respectively.

The crosstalk probability of deep SiPMs is higher than that of shallow SiPMs, owing to the higher PDE and the deeper junction. In fact, the shallow trench isolation (STI) field oxide shields the shallow SPAD avalanche multiplication region [10], i.e., the actual photon emitter. Conversely, the avalanche multiplication region of deep SPADs is well below the STI level [10]. As a result, the deep trench is the only optical barrier that prevents secondary photons from reaching nearby pixels.

The afterpulsing probability has been calculated by the percentage ratio of the sum of afterpulse events to the total number of events. Figure 6b shows the afterpulsing probabilities as a function of excess bias. For all devices the afterpulsing probability is below 1% at 5 V excess bias and is less than 0.3% at an excess bias voltage of 3 V.

### 3.4. Timing Response

**Figure 7:**
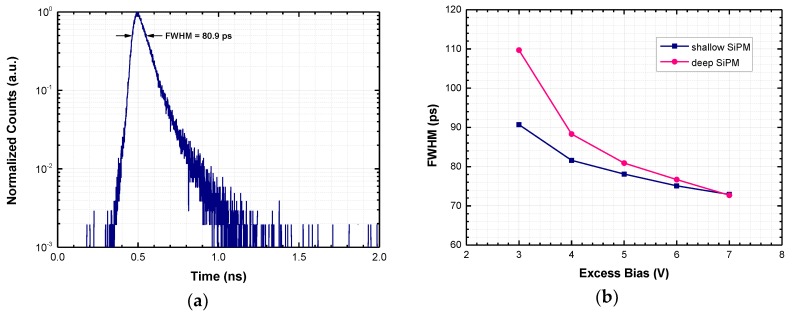
(**a**) Single-photon timing response at 850 nm of a deep SiPM operated at 5 V excess bias; (**b**) Measured timing jitter (FWHM) of the two developed SiPMs as a function of excess bias.

Figure 7a shows, on a log scale and after amplitude normalization, the timing response of a deep SiPM to a pulsed laser (FWHM ~ 40 ps) at 850 nm, which uniformly illuminates the entire sensor area, as acquired by a standard time-correlated single-photon counting (TCSPC) setup. The laser has been attenuated to less than 0.05 detected photons per pulse; as a result, the probability of having more than one photon per pulse is negligible. The timing response is given by the superposition of all single-cell responses. The measured photon-timing jitter, i.e., the FWHM of the photon arrival time distribution, is approximately 80 ps at 5 V excess bias. Figure 7b shows the FWHM dependence on excess bias voltage.

Thanks to the junction isolation from the substrate, the ‘diffusion tail’ in Figure 7a, which is due to minority carriers photogenerated in neutral regions and diffusing toward the space charge, is very fast. Such extremely fast tail leads to approximately 500 ps FW1/100M (full width at 1/100 of the peak), thus being attractive to applications requiring high timing resolution measurements of fast optical signals with wide dynamic range, e.g., in time-resolved NIRS [19].

## 4. Discussion and Conclusions

In this work, we have developed and characterized novel SiPMs in a 0.16 µm–BCD technology. Table 1 summarizes the performance of the fabricated SiPMs and compares it to the best commercially-available SiPMs (e.g., from SensL [20], Hamamatsu [21], AdvanSiD [22], Excelitas [23], Broadcom [24] and Ketek [25]), to the CMOS analog SiPM reported in [7] and to the BCD analog SiPM reported in [8]. 

Despite the lower FF, the peak PDE is comparable to that of the latest commercial SiPMs. However, the DCR density, i.e., the DCR normalized to the sensor area, is higher than what is achieved by the best detectors on the market. Correlated noise is comparable to that of best-in-class SiPMs and delayed crosstalk is negligible. Finally, thanks to the very fast diffusion tail, timing response is among the best reported [26].

This work paves the way for the development of advanced and cost-effective systems-on-chip (SoC), integrating SiPM sensors, analog readout [27], and digital processing electronics.

## Figures and Tables

**Table 1:** Summary of the performance of the developed SiPMs compared to some commercial SiPMs.

Device	Cells	FF (%)	Peak PDE (%)	DCR/area (kHz/mm ^2^)	Crosstalk (%)	Timing Jitter (ps)
SensL C-Series [20]	282	72	35 ^1^	30 ^1^	10 ^1^	NA
Hamamatsu S13360 [21]	667	74	40 ^2^	53 ^2^	3 ^2^	NA
AdvanSiD ASD-RGB1S [22]	625	60	32.5 ^3^	< 100 ^4^	32 ^3^	NA
Excelitas C30742-11 [23]	400	NA	33 ^5^	150 ^5,6^	NA	NA
Broadocom AFBR-S4N44C013 [24]	15,060	76	54 ^7^	170 ^7^	27 ^7^	NA
Ketek PM11 Series [25]	1600	NA	43 ^8^	100 ^8^	20 ^8^	230 ^8^
0.35 µm CMOS SiPM [7]	256	73.7	34 ^9^	584 ^9^	33.5 ^9^	240–340
0.18 µm BCD SiPM [8]	400	NA	1.3	20000	40	NA
BCD Shallow SiPM (this work)	100	53	23 ^10^	890 ^10^	2.8 ^10^	78
BCD Deep SiPM (this work)	100	53	33 ^10^	732 ^10^	9.7 ^10^	81

^1^ V_EX_ = 2.5 V and T = 21 °C. ^2^ V_EX_ = 3 V and T = 25 °C. ^3^ V_EX_ = 4 V and T = 20 °C. ^4^ V_EX_ = 2 V and T = 20 °C. ^5^ V_EX_ = 5 V and T = 25 °C. ^6^ Only the active area is considered. ^7^ V_EX_ = 6.5 V and T = 20 °C. ^8^ V_EX_ = 5 V and T = 21 °C. ^9^ V_EX_ = 6 V and T = 25 °C. ^10^ V_EX_ = 5 V and T = 27 °C.
